# Supplementary material for: Control of cystic echinococcosis in the Middle Atlas, Morocco: Field evaluation of the EG95 vaccine in sheep and cesticide treatment in dogs
Source: PLoS Negl Trop Dis. 2021 Mar 8;15(3):e0009253. doi: 10.1371/journal.pntd.0009253 (PMC7971873; doi:10.1371/journal.pntd.0009253)
Supplement: Attachment — Submitted filename: moroco-plos-review.pdf [file pntd.0009253.s001.pdf]

This is an interesting manuscript describing the outcome of sheep vaccination, dog antihelmintic treatment and combined measures on the prevalence of cystic echinococcosis (CE) in sheep. Generally I think the data on the outcome for sheep vaccination is sound and is a further illustration of the efficacy of the Eg95 vaccine as a tool for controlling CE. However, there are important issues with the study of echinococcus in dogs and the interpretation.
From the description of the data and the methodology and results, the authors only report the changes in prevalence in dogs in Ain Leu 2, which was the group of dogs receiving anthelmintic therapy. They report a significant decline in prevalence between the start of the study (December 2015) and the end of the study (April 2019). One of the issues with that is they only compare 2 time points. In many societies there is an increase in the slaughter of sheep prior to the onset of winter. I do not know if this is the case in Morrocco, but if it is, then dogs are more likely to be infected in December compared to April or August. So seasonal patterns of sheep slaughter may account for some of the variation in prevalence of Echinococcus in dogs. Indeed the authors state that there is no significant difference between April 2016 and April 2019 which is a more appropriate comparison as it would avoid confounding effects due to season slaughter of sheep for example. Of course the hypothesis that the initial treatment in December 2015 reduced the prevalence in dogs and continued treatment every 4 months maintained that low prevalence is also consistent with this observation. I analysed the data that the authors presented by logistic regression and using each time point as a fixed effect I obtained the following result:

```
Coefficients:
                 Estimate Std. Error z value Pr(>|z|)
(Intercept)     -0.6225     0.2643  -2.356  0.01850 *
timepointapr16  -0.9461     0.5581  -1.695  0.09006 .
timepointapr17  -0.6302     0.5330  -1.182  0.23707
timepointapr18  -0.8438     0.5244  -1.609  0.10758
timepointapr19  -1.6462     0.6616  -2.488  0.01284 *
timepointAug16  -2.1501     0.7753  -2.773  0.00555 **
timepointaug17  -1.5747     0.6635  -2.373  0.01763 *
timepointaug18  -2.1808     0.7747  -2.815  0.00488 **
timepointdec16  -1.2338     0.5487  -2.248  0.02455 *
timepointdec17  -1.2493     0.5986  -2.087  0.03688 *
timepointdec18  -1.2870     0.5974  -2.154  0.03121 *
---
Signif. codes:  0 ‘***’ 0.001 ‘**’ 0.01 ‘*’ 0.05 ‘.’ 0.1 ‘ ’ 1
```

This indicates that time points April 2019, August 2016, August 2017, August 2018, December 2016, December 2017 and December 2018 all had lower prevalences than December 2015, the baseline or start of the study. OK, this is consistent with some decrease in the prevalence in dogs. However, using a post hoc analysis (ie correcting for multiple comparisons) we get no significant difference between any two observational periods:

```
$`pairwise differences of timepoint`
 contrast      estimate    SE  df z.ratio p.value
 apr16 - apr17  -0.3159 0.675 Inf -0.468  1.0000
 apr16 - apr18  -0.1023 0.668 Inf -0.153  1.0000
 apr16 - apr19   0.7001 0.781 Inf  0.897  0.9983
 apr16 - Aug16   1.2040 0.879 Inf  1.369  0.9558
 apr16 - aug17   0.6286 0.782 Inf  0.804  0.9993
 apr16 - aug18   1.2347 0.879 Inf  1.405  0.9475
 apr16 - dec15  -0.9461 0.558 Inf -1.695  0.8386
 apr16 - dec16   0.2877 0.688 Inf  0.418  1.0000
 apr16 - dec17   0.3032 0.728 Inf  0.416  1.0000
 apr16 - dec18   0.3409 0.727 Inf  0.469  1.0000
```

apr17 - apr18	0.2136	0.648	Inf	0.330	1.0000
apr17 - apr19	1.0159	0.763	Inf	1.332	0.9635
apr17 - Aug16	1.5198	0.863	Inf	1.760	0.8036
apr17 - aug17	0.9445	0.765	Inf	1.235	0.9786
apr17 - aug18	1.5506	0.863	Inf	1.797	0.7823
apr17 - dec15	-0.6302	0.533	Inf	-1.182	0.9845
apr17 - dec16	0.6035	0.667	Inf	0.904	0.9982
apr17 - dec17	0.6190	0.709	Inf	0.873	0.9987
apr17 - dec18	0.6568	0.708	Inf	0.928	0.9978
apr18 - apr19	0.8023	0.757	Inf	1.060	0.9933
apr18 - Aug16	1.3063	0.858	Inf	1.522	0.9128
apr18 - aug17	0.7309	0.759	Inf	0.963	0.9969
apr18 - aug18	1.3370	0.858	Inf	1.559	0.8993
apr18 - dec15	-0.8438	0.524	Inf	-1.609	0.8789
apr18 - dec16	0.3900	0.661	Inf	0.590	1.0000
apr18 - dec17	0.4055	0.703	Inf	0.577	1.0000
apr18 - dec18	0.4432	0.702	Inf	0.632	0.9999
apr19 - Aug16	0.5039	0.948	Inf	0.531	1.0000
apr19 - aug17	-0.0715	0.859	Inf	-0.083	1.0000
apr19 - aug18	0.5347	0.948	Inf	0.564	1.0000
apr19 - dec15	-1.6462	0.662	Inf	-2.488	0.3112
apr19 - dec16	-0.4124	0.774	Inf	-0.533	1.0000
apr19 - dec17	-0.3969	0.810	Inf	-0.490	1.0000
apr19 - dec18	-0.3591	0.809	Inf	-0.444	1.0000
Aug16 - aug17	-0.5754	0.950	Inf	-0.606	0.9999
Aug16 - aug18	0.0308	1.030	Inf	0.030	1.0000
Aug16 - dec15	-2.1501	0.775	Inf	-2.773	0.1683
Aug16 - dec16	-0.9163	0.873	Inf	-1.049	0.9938
Aug16 - dec17	-0.9008	0.905	Inf	-0.995	0.9960
Aug16 - dec18	-0.8630	0.905	Inf	-0.954	0.9972
aug17 - aug18	0.6061	0.949	Inf	0.639	0.9999
aug17 - dec15	-1.5747	0.663	Inf	-2.373	0.3839
aug17 - dec16	-0.3409	0.776	Inf	-0.440	1.0000
aug17 - dec17	-0.3254	0.812	Inf	-0.401	1.0000
aug17 - dec18	-0.2877	0.811	Inf	-0.355	1.0000
aug18 - dec15	-2.1808	0.775	Inf	-2.815	0.1521
aug18 - dec16	-0.9471	0.873	Inf	-1.085	0.9920
aug18 - dec17	-0.9316	0.905	Inf	-1.030	0.9947
aug18 - dec18	-0.8938	0.904	Inf	-0.989	0.9962
dec15 - dec16	1.2338	0.549	Inf	2.248	0.4700
dec15 - dec17	1.2493	0.599	Inf	2.087	0.5867
dec15 - dec18	1.2870	0.597	Inf	2.154	0.5378
dec16 - dec17	0.0155	0.721	Inf	0.022	1.0000
dec16 - dec18	0.0532	0.720	Inf	0.074	1.0000
dec17 - dec18	0.0377	0.759	Inf	0.050	1.0000

Results are given on the log odds ratio (not the response) scale.
P value adjustment: tukey method for comparing a family of 11 estimates

So this is not looking so good. If the dog treatment failed to have an influence on the prevalence in dogs, then it is not so surprising that it failed to have a synergistic effect with the vaccination in sheep to reduce further the prevalence in sheep.

Finally using time point as a continuous variable (ie 0-40 months after the start of the study), does show there is an overall reduction in prevalence over time indicating treatment of dogs may be having an effect… but the problem is that you do not have equal numbers observations for December, April and August to eliminate potential effects of confounding due to season transmission.

```
Coefficients:
               Estimate Std. Error z value Pr(>|z|)
(Intercept) -1.08014    0.21375  -5.053 4.34e-07 ***
time        -0.03379    0.01111  -3.041  0.00236 **
---
Signif. Codes:  0 ‘***’ 0.001 ‘**’ 0.01 ‘*’ 0.05 ‘.’ 0.1 ‘ ’ 1
```

OK, this indicates dog prevalence may have decreased over time. Finally I removed the last two data points (December 2018 and April 2019) so there was no imbalance with some seasons having more data points and added a random effect(ie some unknown random factor effecting the prevalence at each time point). This resulted in:

```
Random effects:
 Groups    Name        Variance Std.Dev.
 timepoint (Intercept) 0.1043   0.323
Number of obs: 9, groups:  timepoint, 9

Fixed effects:
               Estimate Std. Error z value Pr(>|z|)
(Intercept) -1.15017    0.33009  -3.484 0.000493 ***
time        -0.03666    0.01857  -1.974 0.048376 *
---
Signif. codes:  0 ‘***’ 0.001 ‘**’ 0.01 ‘*’ 0.05 ‘.’ 0.1 ‘ ’ 1
```

This suggested that there was a (just) significant decrease in prevalence in dogs over time following the start of anthelmintic treatment. This final analysis is, in my opinion, the most robust analysis of the dog data and does indicate a possible decrease, albeit the significance is marginal.

In summary, the data as presented does not conclusively show that the anthelmintic treatment of dogs actually had an effect. It may have done, but in terms of statistical evidence I think it is, at best, marginal. The reasons may be that there were many dogs that avoided treatment and hence no decrease in prevalence was observed. Or that a decrease in prevalence did occur but was not sufficiently large to be detected or was hidden by confounders. Or if there was a variation in prevalence in dogs it was not caused by the anthelmintic treatment. Or 4 monthly treatments are insufficiently frequent to have an effect. So even if you use the final analysis, you still have the problem of not knowing what would have happened if dogs had not been treated...
Can this problem be fixed? Yes if they have data for dogs in Ain Leu 1 where the dogs were not treated. This could serve as controls and comparing changes in prevalence between those two groups (Ain Leu 1 and Ain Leu 2) would indicate if anthelmintic treatment was responsible for any changes in prevalence in dogs or other, not necessarily known factors. If this data is not available, then the authors need to discuss this as a weakness in the study and discuss the fact that any reduction in prevalence in dogs is not statistically convincing (or at best marginal). Also that due to

the lack of available controls, the possible change in prevalence in dogs may be due to something other than dog treatments. Hence this may be the reason there was no observed synergistic effect when dog treatment was combined with vaccination of sheep.

Other issues
Please avoid using statements such as:
“The prevalence and abundance of dead cysts was higher in vaccinated sheep than non-vaccinated sheep, but this was not statistically significant.”
Frequentist statistics usually test the null hypothesis, ie that there is no difference between vaccinated and non vaccinated sheep. If the p value is <0.05 (by convention), there is assumed to be sufficient evidence to reject the null hypothesis and conclude there is a difference. So the statement is non nonsensical to say there is a difference but it is not significant. The correct way of reporting should be “There was insufficient statistical evidence to conclude there was a difference in the prevalence and abundance of dead cysts in vaccinated sheep compared to non-vaccinated sheep.”

Paul Torgerson
