# Supplementary material for: Control of cystic echinococcosis in the Middle Atlas, Morocco: Field evaluation of the EG95 vaccine in sheep and cesticide treatment in dogs
Source: PLoS Negl Trop Dis. 2021 Mar 8;15(3):e0009253. doi: 10.1371/journal.pntd.0009253 (PMC7971873; doi:10.1371/journal.pntd.0009253)
Supplement: Attachment — Submitted filename: 2101 Reply to reviewer comments.docx [file pntd.0009253.s002.docx]

14 December 2020

Dear Professor Basáñez, Associate Editor and Professor Jokelainen, Deputy Editor

PLOS NTD

Thank you for your and the reviewers’ comments on our manuscript. We are grateful to the reviewers who read comprehensively our manuscript and made very constructive suggestions. We tried to address each of the issues raised in a revised manuscript and briefly discussed more important topics below.

Sincerely,

Ms Fatima Amarir, on behalf of all co-authors

**Reviewer 1**

Include information on sheep management: The manuscript has been amended in lines

- Add more detail on study design: Further details of the study design have been added in lines 118-121, 156-157. Additional data was provided in the text rather than being added to Table 1.
- How many animals does each producer have? Among the 32 breeders recruited in the study, on average they have 100 to 250 sheep per farm approximately. This information has been added (M& M section).
- Can the proportion be estimated of animals that entered the study in relation to the total number of dogs and sheep in the work areas? We are unable to provide precise data because the number of sheep and dogs and in the region is not known. It can be mentioned that around 90% of owned dogs were treated in the location where chimioprevention of dogs was performed. In a previous study, performed in the Middle Atlas, owned dogs accounted for 2/3 or the canine population.
- What is the frequency with which a farmer slaughters animal that can infect dogs? Farmers generally slaughter their animals twice a year, on the occasion of religious feast day (the feast of sacrifice) and during the sheep-shearing ceremony in the late spring. Otherwise, they may slaughter their animals at occasions such as birth, baptism, death,…). This information was clarified in lines 136-137.
- Were US diagnoses done at the beginning? US was not done prior to vaccination as young lambs were assumed free of *E.granulosus*. This was clarified in line 215.
- Now were dogs selected, what PCR test? All owned dogs were selected but we failed to catch them all. Copro-PCR test used was further detailed in line 210.
- How the dogs to be studied were selected by the arecoline test? Dogs are selected for the arecoline test according to the possibility of catching them so it is based on the behaviour of the dog.
- Were they evaluated in each farm or were pools made? The dogs were evaluated individually in each farm and no pool was made (specified in line 209).
- How were the sheep selected for US or necropsy? The assessment method depended mostly on the decision of the owner: when slaughtering was not allowed, ultrasound was performed (clarified in line 166-167).
- Sheep with US and sheep euthanized: are the two diagnostic systems given the same value? Comparison of the diagnostic performance of necropsy and US. Necropsy and US data are mutually exclusive. In a separate study (to be published) we found high sensitivity and specificity of US compared to necropsy in the study area (thin animals). In the current study, increased precision due to a larger number of observations when adding US data compensated the potential lack of US accuracy. It was not possible to compare US and necropsy data in the framework of this study. We tried to better describe this in the manuscript.
- Tables presenting US and necropsy data: we opted to present necropsy data on their own because dissection is the gold standard. Unfortunately, estimates were unprecise due to a rather low number of observations. This is why we decided to present data including both US and necropsy results (parallel test combination). Though, US data on its own could be made available, we feel this would not be so relevant (lack of both precision and accuracy) and would fill even more our tables. In our statistical analyses, we assured that the diagnosis effect was not significant (reemphasised in the text). As indicated above, a comprehensive evaluation of the US is foreseen in a separate article.
- Table 4. testing pre or post treatment: There is no significant variation of prevalence in dogs after the first treatment. Therefore, a mean was calculated before (35%) and after (12%) the first treatment. We tried to clarify this in the table.
- Discussion – include comparison of the burden on infection: These data are included in the discussion lines 421-422.
- Cyst fertility: Individual fertility of cysts was not assessed. Viability was assessed on the visual aspect of cysts at necropsy or US.
- Who dewormed the dogs: This was done by the researchers, clarified in lines 208-209.
- Explain why high prevalence remained in dogs: an average of 12% after 4 months corresponds to a mean prevalence of 6%, assuming that deworming is 100% effective. This value is not surprising and corresponds to a monthly incidence of 4%, as observed in other studies. We tried to address this in lines 463-466.
- When are sheep slaughtered? Sheep were slaughtered at the end of the study, when ewes were about 4-year-old. Clarified in line 165.
- Effects on infection rates in children: Our study failed to demonstrate that 4 monthly treatment of owned dogs had any effect on incidence in sheep. Yet, more frequent treatments or coverage of the whole canid population (logistically complicated) could prove effective. In addition, we agree that treating dogs in close contacts with humans is recommended to reduce the risk of transmission to people. We tried to incorporate these important considerations in the discussion (line 507).
- Final summary table. We declined the proposition to create an additional table because of the already high number of tables in the manuscript. We hope that the clarifications we made in the text are sufficient. If not, we would be prepared to follow the editor’s recommendations and make an additional table as requested.

**Reviewer 2**

- We deeply appreciate the analytical work of Reviewer 2 and agree with his findings. We acknowledge that the study design was not optimal, particularly with the dog treatments. Ideally, all dogs (and not only owned dogs) should have been included, repetitions in different sites should have been implemented etc. for a comprehensive analysis of the effect of dog chemotherapy. As a consequence, our work should not be seen as contradicting previously published work and models but should illustrate how heavy and difficult it is to implement a control strategy based on dog chemotherapy. We tried to incorporate these nuances in the discussion.
- We followed the recommendation of reviewer 2 to check a possible seasonal effect on dog incidence, which we did not find significant. We only found significant the difference between the prevalence before and after the first treatment, indicating that dog chemotherapy had an effect on prevalence in dogs. However, the high incidence in the study area causes reinfestation reaching 12% on average after 4 months, roughly corresponding to a mean prevalence of 6%, assuming that chemotherapy is 100% effective (0+12)/2. We also incorporated these considerations in the revised manuscript.
- Finally, we accept the remark about the non-significance of differences and made appropriate changes throughout the document.
